# Formation Mechanism and Influencing Factors of Micro-Nano Dual-Size NiPd Alloy with a Flake-like Microstructure

**DOI:** 10.3390/ma18040829

**Published:** 2025-02-14

**Authors:** Hui Zhao, Wuyun Xiao, Dahai Liu, Wei Zhao, Kang Zhou, Linghui Hou, Xinhua Ma, Yanxia Liang

**Affiliations:** State Key Laboratory of NBC Protection for Civilian, Beijing 102205, China; nicholas_chao05@163.com (H.Z.); xiaowuyun@sklnbcpc.cn (W.X.); l_dahai@126.com (D.L.); zhaoxiao_yu1995@163.com (W.Z.); 13588030692@163.com (K.Z.); hlh@whu.edu.cn (L.H.); coralhua@126.com (X.M.)

**Keywords:** high-energy ball milling, NiPd alloy, flake-like microstructure, dual-size nanocrystalline, ball-to-powder ratio

## Abstract

The large-scale and engineering preparation of NiPd powder with an excellent structure is crucial for promoting the development of catalytic applications and the utilization of hydrogen energy. In this study, micro-nano dual-size NiPd alloy with a flake-like microstructure was synthesized using a simple and efficient high-energy ball milling method. Micro-structural analysis was performed on samples at various stages of ball milling to investigate the atomic migration and refinement mechanisms during the process. The microstructure and elemental distribution at different stages of particle refinement were examined, and the particle size distribution of NiPd alloy at varying ball milling times was statistically analyzed. The NiPd alloy produced using this method exhibits two distinct structural features: a flake-like polycrystalline structure and a dual-size nanocrystalline structure. A flake-like polycrystalline powder was achieved using relatively short ball milling durations and characterized by a polygonal two-dimensional structure with a single particle aspect ratio of 24. After 60 h of ball milling, the NiPd alloy developed a dual-size nanocrystalline structure. Under a high ball-to-powder ratio, the average particle size of the alloy decreased to 3.6 μm, more than tenfold smaller than the initial size. The alloy particles transformed from their original elemental metals into alloy phases, with nanoscale alloy particles (approximately 158 nm) adhering to micrometer-sized alloy particles. Additionally, the influence of the ball-to-powder ratio on the refinement process and outcomes was investigated, indicating that a higher ball-to-powder ratio can effectively improve preparation efficiency by accelerating the ball milling process and reducing the final particle size of the NiPd alloy at each milling stage.

## 1. Introduction

The structure of metal powders significantly influences their physical and chemical properties. Flaky metals possess a unique structure characterized by a high aspect ratio, which enhances material adhesion, improves shielding effects, and boosts electrical conductivity. Nickel (Ni), as an industrial material, is widely utilized in battery manufacturing, tribology, surface science, and nanoscience due to its superior physicochemical properties [1,2,3]. Among these applications, nickel-based catalysts exhibit excellent performance in terms of high efficiency, selectivity, cost-effectiveness, and multifunctionality. Compared to precious metals like Pt and Pd, nickel-based catalysts are more economical while demonstrating significant potential in methane conversion, carbon dioxide reduction, diesel steam reforming, and hydrogenation reactions [4,5,6,7]. To further enhance catalytic performance, alloying pure nickel with other elements such as cobalt (Co) or molybdenum (Mo) to produce NiCo and NiMo alloys is an effective approach. These alloys have garnered extensive attention and research in electrocatalysis, chemical reactions, and energy conversion [8,9,10,11,12,13]. NiPd alloy, known for its exceptional properties, finds applications not only in catalysis but also in electrochemistry and solar cells [14,15,16]. Kamarulzaman et al. prepared NiPd-reduced graphene oxide (rGO) films using liquid-phase deposition and investigated the impact of Pd content on photovoltaic parameters, revealing that device performance is highly sensitive to Pd concentration [17]. Another strategy to improve material performance is through nanosizing. NiPd nanocrystals embedded in nitrogen-doped graphene were synthesized by pyrolyzing Ni and Pd salts along with other mixtures, and the products exhibit remarkable stability and methanol tolerance in oxygen reduction reactions [18]. Huang et al. developed NiPd alloy nanoparticles supported on ZnO using plant-mediated synthesis for selective hydrogenation reactions. The catalytic efficiency of the alloy was found to be 1.9 times higher than that of Pd/ZnO [19]. From a preparation perspective, most nanoalloy materials mentioned above are obtained through methods such as pyrolysis, vapor deposition, and chemical reduction. However, these processes are relatively complex and time-consuming. To achieve large-scale production, alternative synthesis methods need to be explored.

High-energy ball milling (HEBM) is a method for preparing polycrystalline alloy materials using physical collision, friction, and other mechanical actions. Due to its high strain and deformation rate, HEBM can produce nanocrystalline powders and other advanced materials. This technique enables the synthesis of stable and metastable phases in various alloy systems, including intermediate phase, solid solution, and amorphous alloy. The advantages of HEBM include simplicity, broad applicability, and cost-effectiveness. Compared to conventional ball milling, HEBM provides higher input energy, making it highly suitable for preparing metal samples with diverse structural types, particle sizes, and nanostructures [20,21,22,23]. Abbas et al. obtained AM60 magnesium alloy with a grain size of 14 nm using HEBM [24]. The transition metal vanadate was prepared for enhancing lithium storage performance. Cobalt vanadate oxide exhibited excellent electrochemical properties, offering an effective approach for advanced electrode material fabrication [25]. HEBM has been shown to improve alloy performance across various applications [26], and current research is increasingly focusing on multi-phase materials rather than dual-phase materials. Ball milling parameters significantly influence the final product’s characteristics. For instance, Hafs et al. synthesized nanocrystalline Fe_60_Al_35_Mg_5_ powder using HEBM, and the product exhibited soft ferromagnetic properties with magnetic parameters highly sensitive to ball milling duration [27]. Fe_30_Co_20_Ni_20_Mn_20_Al_10_ alloy with a FCC crystal phase was prepared using HEBM. Studies on the changes in phase, microstructure, morphology, and magnetism over time revealed that the magnetic properties were closely related to microstructural features [28]. HEBM can be effectively utilized for the industrial preparation of nanoparticles with high catalytic activity. Zhang et al. demonstrated that under normal pressure, ball milling controlled the strong metal–support interaction of partially oxidized TiC loaded with Pd nanoparticles, promoting electron transfer [29]. Extensive studies illustrate that HEBM is a convenient and efficient method for nanoparticle preparation. However, detailed research on the preparation of NiPd alloy using HEBM remains limited. To address the challenges of low yield and the complex processes associated with the chemical synthesis of NiPd nanoparticles, it is imperative to investigate the influencing factors of high-energy ball milling for NiPd alloy.

In this work, we successfully prepared NiPd alloy with flaky polycrystalline and dual-size nanocrystalline structures by optimizing the HEBM parameters. The radial dimensions of the two-dimensional flaky polycrystalline particles ranged from 1 µm to 71 µm, while the average particle size of the dual-size nanocrystalline NiPd was 3.6 µm, with smaller particles of approximately 158 nm attached to the peripheries. The powder is roughly spherical shape with a flaky morphology and thickness in the hundreds of nanometers. Relevant parameters during the preparation were explored accordingly. Scanning electron microscopy (SEM), energy dispersive spectroscopy (EDS), and X-ray diffraction (XRD) were employed to perform characterization of the samples at different stages, elucidating the morphological and compositional process at each stage of ball milling and showing the impact of ball milling time on the samples. Additionally, the effect of the ball-to-powder ratio (BPR) on the HEBM process was examined. Our results indicate that both ball milling time and BPR significantly influence the resultant particle size and processes involved in the NiPd ball milling system. This study demonstrates that HEBM can effectively control the structure, morphology, grain size, and alloying degree of NiPd alloys, thereby providing a new pathway for its fabrication to fulfill their wide applications.

## 2. Experiment

The purity of the Ni and Pd powders used in this experiment was higher than 99.9%, as indicated in the accompanying reports. Both Pd (ZhongNuo Advanced Material (Beijing) Technology Co., Ltd., Beijing, China) and Ni (ZhongYan Precise Instrument (Beijing) Technology Co., Ltd., Beijing, China) powders were commercially sourced. Specifically, the Pd powder was spherical with an original particle size of 325 mesh, while the Ni powder was dendritic and sieved with an original particle size of 350 mesh. HEBM experiments were conducted using the Fritsch P-7 enhanced planetary ball mill (Fritsch, Idar-Oberstein, Rheinland-Pfalz, Germany) from Germany. Prior to conducting HEBM, the required masses of grinding balls and elemental powders were calculated based on the ball-to-powder ratio and atomic ratio. In this study, the ball-to-powder ratios were 10:1 and 20:1, with an atomic ratio of Ni:Pd = 10:1 for the initial powders. Based on these calculations, 63 g and 66 g of grinding balls were weighed and placed into grinding vials 1 and 2, respectively. Subsequently, 5.3308 g of Ni and 0.9677 g of Pd were added to vial 1, while 2.7985 g of Ni and 0.5061 g of Pd were added to vial 2. Finally, 6 mL of ethanol was added to each vial. The lids were securely tightened, and the vials were placed in the ball mill for processing. All ball milling processes were performed at room temperature (about 25 degrees). The ball milling parameters were set as follows: the rotation speed was 500 rpm. To ensure optimal instrument operation and minimize potential thermal effects on the samples, the ball milling process was carried out intermittently. Specifically, after 6 min of milling, the process was paused for 4 min of air cooling. The ratio of the self-rotation to the orbital rotation of the grinding vials was set to 1:2. Both the grinding vials and balls were made of tungsten carbide (WC), with the grinding balls having a diameter of 5 mm. Prior to formal ball milling, pre-ball milling was conducted with the same atomic ratio to ensure that the grinding balls were fully coated with the materials, thereby effectively reducing impurity content. To enhance ball milling efficiency and reduce adhesion to the vials’ walls, wet ball milling was employed using chromatographic-grade ethanol with a purity exceeding 99.997% (Aladdin, Shanghai Aladdin Bio-Chem Technology Co., Ltd., Shanghai, China) as the process control agent. Sample and ball weighing, vial filling, loading, and sampling at various time were performed in a glove box (Etelux Lab2000, Etelux Inertgas system (Beijing) Co., Ltd., Beijing, China) under a nitrogen atmosphere with a purity greater than 99.999%. In addition, ball milling was carried out under a nitrogen atmosphere, thus ensuring its separation from water and oxygen contamination.

During the experiment, the ball milling time was strictly controlled. At designated intervals, the ball milling vials were transferred to the glove box for sampling, and the samples were stored in chromatographic ethanol. Characterization of the microstructure and phase structure was conducted using SEM (ZEISS, Baden-Württemberg, Germany), EDS (Oxford, Oxford, UK), and XRD (Bruker, Billerica, MA, USA). Particle size statistics were obtained using Nano Measurer 1.2 software, with the Martin diameter (the length of the area bisector of polygonal particles in the horizontal measurement direction) serving as the particle diameter. SEM images were analyzed using the software, and particle size statistics were performed on all particles within selected areas. By comparing statistical results from different sample numbers, it was found that the maximum deviation in results between sample numbers ranging from 100 to 600 was within 6%. In this study, a sample number of 200 was chosen, and data statistics were obtained by analyzing all particles in a specific area to generate the particle size distribution histogram.

## 3. Results and Discussion

### 3.1. Characterization Results and Analysis of the Original Powder

The powders used in this study included micron-sized Ni and Pd, both with a purity of 3N. To understand their microstructure and phase composition, it was essential to characterize these powders before the HEBM experiment, obtaining baseline data such as particle size for comparison with post-milling samples. The following section presents the characterization results of the initial Ni and Pd powders.

**Figure 1:**
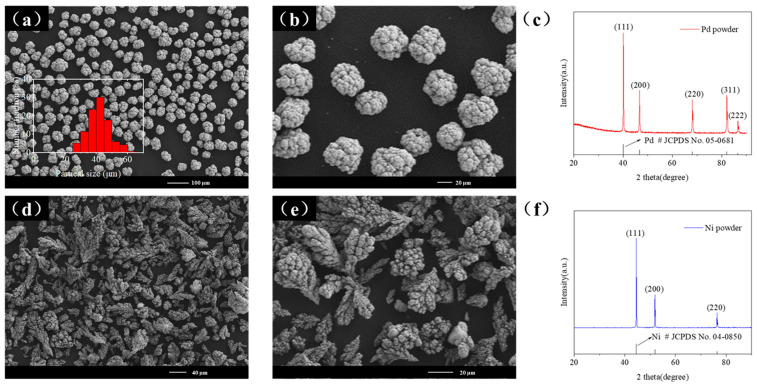
(**a**,**b**) SEM images and (**c**) XRD pattern of the original Pd powder. (**d**,**e**) SEM images and (**f**) XRD pattern of the original Ni powder.

Figure 1a,b show the SEM images of the Pd powder. The particle size distribution suggests a Gaussian distribution, with over 88% of the particles having a size less than 50 μm and an average particle size of 41.74 μm. The Pd powder exhibits an overall spherical morphology. This is primarily due to the fact that commercially available palladium powder is predominantly prepared using atomization and chemical reduction techniques, both of which favor the formation of spherical particles. Additionally, the inherent physical and chemical properties of palladium further enhance its tendency toward spheroidization. The spherical surface exhibits crisscrossing grooves, which can be attributed to the liquid-phase reduction process used to prepare the micron-sized palladium powder. This preparation method results in a large specific surface area, leading to an uneven pattern of the surface morphology. Figure 1c displays the XRD pattern of the Pd powder. Comparison with the standard PDF card confirms that the peak positions match precisely, indicating a polycrystalline structure with the (111) plane as the main crystal plane. Figure 1d–f present the SEM images and XRD pattern of the Ni powder, respectively. The Ni powder exhibits strong magnetism and is typically prepared using electrolysis and chemical reduction methods. During electrolysis, the non-uniform distribution of the electric field and ion diffusion cause Ni powder to grow rapidly in specific directions, leading to dendritic structures. In the chemical reduction process, dendritic structures form due to non-uniform nucleation and growth. Consequently, under an electron microscope, the Ni powder appears as dendritic, irregularly shaped particles, which poses significant challenges for precise particle size measurements. However, from the SEM image, it is evident that the length of the dendritic branches is comparable to the average particle size of the Pd powder. The XRD pattern also confirms a polycrystalline structure with multiple orientations. For HEBM experiments, we selected the most commonly available shapes and sizes on the market to investigate the refinement and alloying processes. The spherical morphology of Pd facilitated uniform mixing, while the irregular dendritic structure of Ni enhanced fragmentation, both of which are advantageous for the HEBM process. Additionally, both powders had an average particle size of approximately 40 µm, which is considered moderate for HEBM and ensures optimal milling efficiency. Therefore, the selection of these original powders was well-suited for this study.

### 3.2. Results and Analysis of the Ball Milling Process

**Figure 2:**
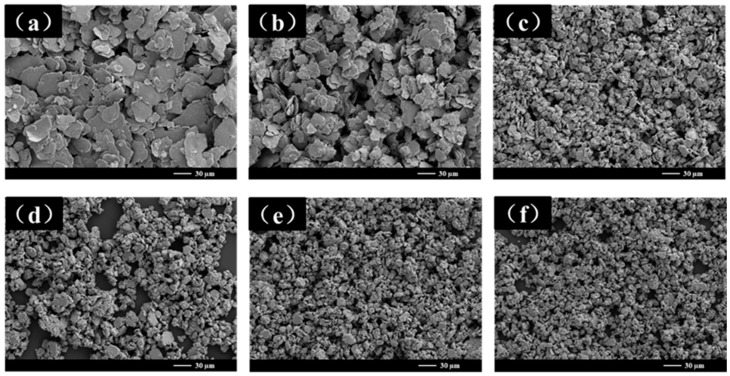
SEM images of NiPd samples with different ball milling times: (**a**) 10 h; (**b**) 20 h; (**c**) 30 h; (**d**) 40 h; (**e**) 50 h; (**f**) 60 h.

Figure 2 shows the SEM images of the samples at different stages during the entire ball milling process with a ball-to-powder ratio (BPR) of 10:1. These samples are denoted as NiPd-10 to NiPd-60 for milling times ranging from 10 h to 60 h. As shown in the figure, the particle size continuously decreases as the ball milling time increases. At 10 h and 20 h (Figure 2a,b), the samples exhibit a typical two-dimensional sheet microstructure, characterized by an irregular polygonal surface. The diameter ranges from 1.76 μm to 70.85 μm, with aspect ratios of 59 and 24, respectively. From 30 h to 50 h (Figure 2c–e), the two-dimensional sheet structure gradually disappears, giving way to smaller particles with increased thickness. The aspect ratio of the samples decreases to 6 (NiPd-50). Figure 2f shows the SEM image of the sample after 60 h of ball milling. Under these conditions, the microstructure exhibits the coexistence of dual-scale morphological features, namely, plate-like structures with an average thickness of approximately 0.8 μm. The micro-sized particles have an average size of 4.63 μm, and their surfaces are attached to numerous nanoparticles, with the smallest particle size being 370 nm. Compared to the original powders, the average particle size of smallest ones has decreased by approximately 100 times. However, due to the significant reduction in particle size, the sample exhibits noticeable agglomeration, as evident in the SEM image at 60 h. Further structural details of the samples will be discussed in subsequent sections along with an analysis of the ball milling process and the fragmentation mechanism.

**Figure 3:**
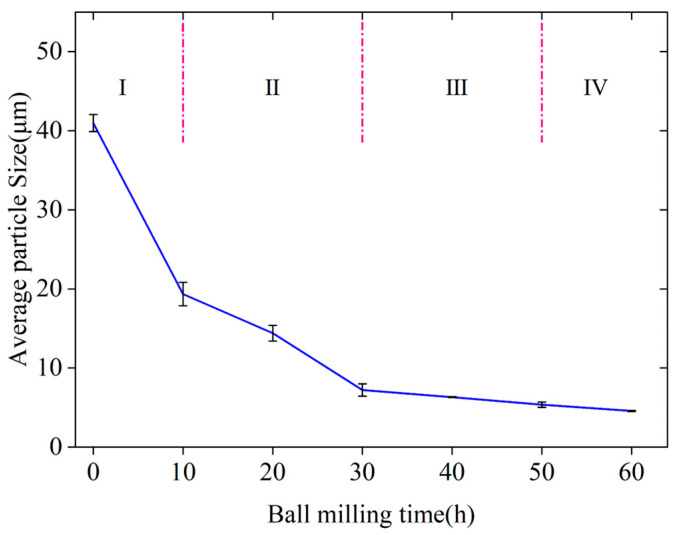
The average particle sizes of NiPd samples varying with ball milling time.

The particle size of NiPd samples at different ball milling stages was statistically analyzed. Figure 3 illustrates the relationship between the average particle diameter of NiPd samples and the ball milling time. The results show a continuous decreasing trend in particle size until equilibrium is reached throughout the ball milling process. Based on the particle size change trend and microstructure characterization results shown in Figure 2, the ball milling process can be divided into four distinct stages as depicted in Figure 3. Stage I (0–10 h): At the beginning of ball milling, large particles are fractured into smaller ones under the impact of grinding balls. Simultaneously, these smaller particles become thinner due to the impact force and weld together with other particles, forming two types of sheet structures: elemental and pre-alloyed particles. This stage is characterized by significant differences in particle size. Stage II (10 h–30 h): As ball milling continues and energy input increases, pre-alloyed and elemental particles that have not yet alloyed gradually coalesce under the combined effects of impact and friction forces. The axial thickness of the particles gradually increases, marking the onset of alloying. Stage III (30 h–50 h): With further increases in ball milling time, mechanical friction causes small fragments to detach from the sheet structures, fostering the formation of grooves and holes on the surfaces of larger particles, making the surfaces rough. The sizes of flake-like particles continuously decrease, and these particles gradually become more circular. Stage IV (>50 h): After 50 h of ball milling, the particle size fluctuates within a certain range and no longer shows a significant reduction. After reaching the critical shear stress provided by the ball milling system, the breaking and combination of NiPd particles reach equilibrium. Further reductions in particle size become increasingly difficult, resulting in size fluctuations within a specific range.

**Figure 4:**
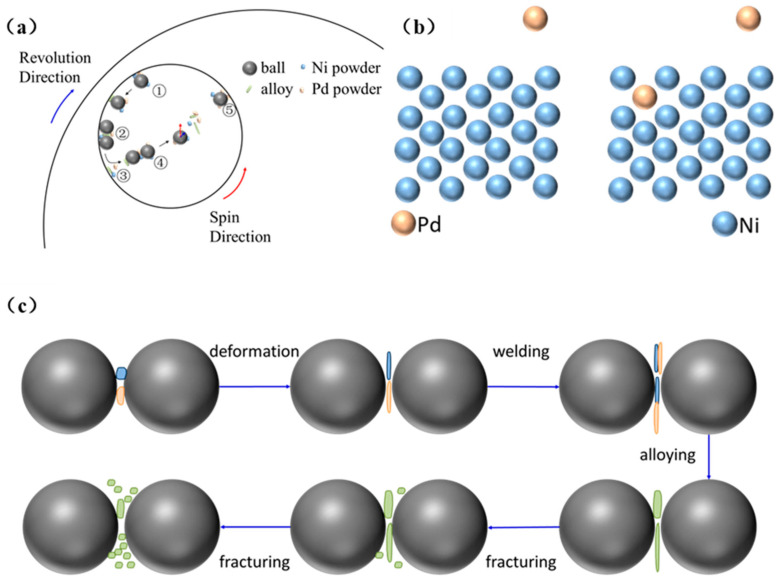
(**a**) Schematic diagram of the movement of the vials, powders, and grinding balls. (**b**) Schematic diagram of atomic migration. (**c**) Schematic diagram of particle morphology changes. The gray balls, blue balls, and orange balls represent grinding balls, Ni atoms, and Pd atoms, respectively. The blue particles, orange particles, and green particles in (**c**) represent Ni powder, Pd powder, and alloyed powder, respectively.

To further investigate the mechanism of particle breakage, the movement and force conditions of the balls and materials during the ball milling process were analyzed. Figure 4 provides a schematic illustration of the sample’s ball milling process. Figure 4a represents the schematic diagram of the movement of the vials, powders, and grinding balls. Their relative motions can be classified into five types, representing the frictional and impact forces from diverse directions that the sample undergoes during the ball milling process. These include frictional forces between the vial wall and grinding ball, between the grinding balls (① and ② in Figure 4a), and from the vial wall (③ in Figure 4a) as well as the impact forces between the grinding balls (② and ④ in Figure 4a) and between the grinding balls and the vial wall (⑤ in Figure 4a). Under the influence of these forces, Pd atoms begin to migrate. Figure 4b,c depict the microscopic changes in atoms and particles. Figure 4b illustrates one mechanism by which Pd atoms diffuse into the Ni interior, replacing Ni atoms to form an alloy. Figure 4c shows the changes at the particle level during the ball milling process, including deformation, welding, and fracturing stages. The microscopic structure of each stage will be explained in detail below. After these stages, alloy powders with a micro- to nano-scale size and flake-like structure are formed.

**Figure 5:**
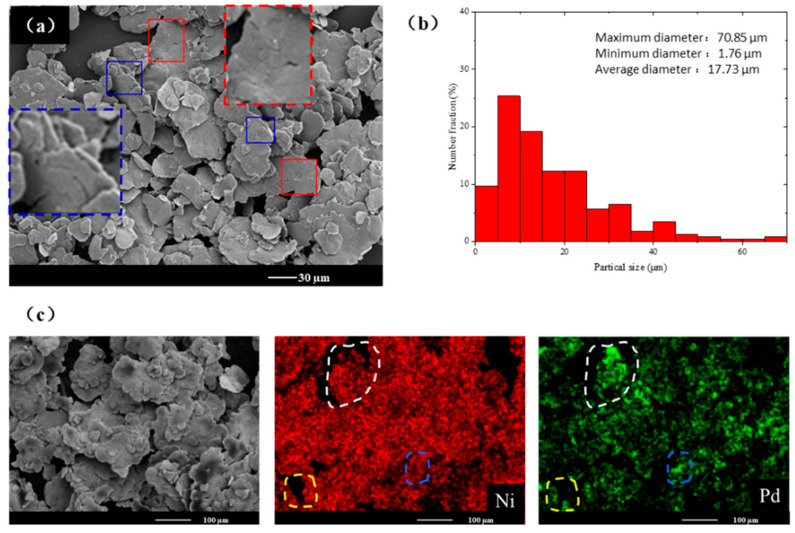
Experimental results of the NiPd-10 sample. (**a**) SEM image. Different color boxes represent different morphologies of particles, and the corresponding boxes with dashed lines are the magnified images of the spots denoted by the same color. (**b**) Particle size distribution histogram. (**c**) Element distribution map, where the irregular dotted boxes of different colors are the regions of the corresponding sample particles.

The previous section provided a brief analysis of the changes in particle size and morphology during the ball milling process. This section will elaborate on the morphology of the sample at each stage and changes observed over time. Figure 5 shows the SEM-EDS images and particle size distribution histogram of NiPd-10. As shown in Figure 5a, the sample exhibits a flaky structure with a relatively smooth surface and an irregular polygonal shape. A significant size difference exists among the particles. As a result, many radial welds (denoted by red boxes) and axial superimpositions (indicated by blue boxes) are visible. Figure 5b presents the particle size distribution histogram for all particles within a selected area. The maximum and minimum particle sizes are 70.85 μm and 1.76 μm, respectively, and the average particle size is 17.73 μm. Particles less than 45 μm in size account for over 96% of the entire grain population. Figure 5c shows an electron image and element distribution map of the sample at this stage. The red and green image represent the distribution of Ni and Pd elements, respectively. The irregular dashed boxes of different colors correspond to individual particles. The content of Ni is higher than that of Pd in the sample, and the distribution of the two elements is non-uniform at the beginning of the ball milling process.

At this stage, the particles primarily undergo welding and deformation. Under the extrusion and impact forces illustrated in Figure 4a, individual particle becomes thinner in the axial direction and larger in the radial direction, leading to sample fragmentation and the generation of smaller particles. Simultaneously, a welding neck occurs between different particles, as shown in the red box of Figure 5a. Additionally, the smaller flaky samples produced under the action of impact force and the particles welded start to coalesce axially, resulting in a significant increase in axial thickness. However, due to the relatively short ball milling process, the insufficient energy input results in loose welding. Both these changes prompt atomic migration, preparing the sample for alloying. As shown in Figure 5b, the particle size distribution of the sample ranges widely from 1 µm to 71 µm. This wide distribution is attributed to the predominant processes of deformation and welding, with minor fragmentation and shedding. The element distribution in Figure 5c indicates that Pd is mainly distributed on the periphery and corners of the flaky particles, while Ni occupies the bulk of the flaky structure. This irregular distribution arises because Pd was added in small amounts in the original material, adhering to Ni powder, beginning to fuse under mechanical force, and initiating the atomic migration.

**Figure 6:**
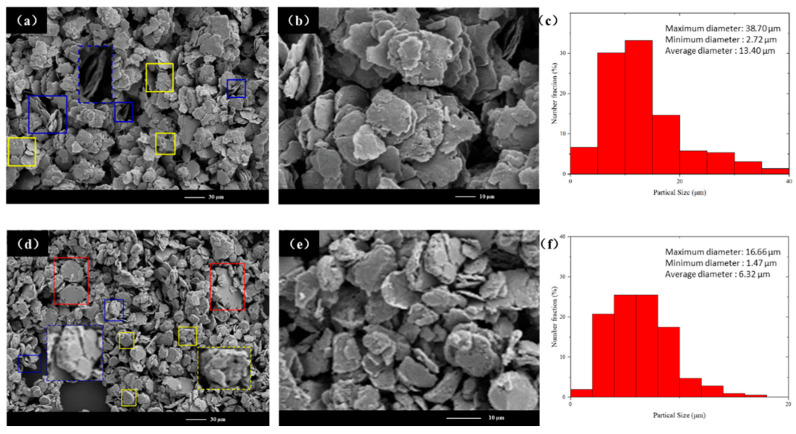
(**a**) Low-magnification SEM image and (**b**) high-magnification SEM image of NiPd-20. (**c**) Statistical graph of NiPd-20 particle size. (**d**) Low-magnification SEM image and (**e**) high-magnification SEM image of NiPd-30. (**f**) Statistical graph of NiPd-30 particle size. The blue box represents the typical radial welding morphology of the particles. The yellow box represents the typical shear friction morphology of the particles, and the red box represents the typical welding morphology of the particles. The dashed boxes with the same color as the respective solid boxes represent their correspondent magnified images.

Figure 6 shows the SEM images and particle size distribution histograms of NiPd-20 and NiPd-30. At this stage, the axial superposition of flaky sample particles becomes more pronounced. Figure 6a,b are low-magnification and high-magnification SEM images of NiPd-20, respectively. Typical axial superpositions (indicated by the blue box in Figure 6a) and changes in particle surface roughness (indicated by the yellow box in Figure 6a) are evident. Figure 6c represents the particle size distribution of NiPd-20, with a particle size ranging from 2.72 µm to 38.7 µm and an average particle size of 13.4 µm. Figure 6d,e are low-magnification and high-magnification SEM images of NiPd-30, respectively. Compared to NiPd-20, the axial superposition of particles becomes more pronounced with increased ball milling time, leading to a further increase in particle thickness (indicated by the blue box in Figure 6d) and significantly increased surface roughness (indicated by the yellow box in Figure 6d), including grooves and a few holes. This contrasts sharply with the large smooth particles produced in the earlier welding and deformation stage. However, few large particles formed in the previous stage still remain (indicated by the red box in Figure 6d). Figure 6f shows the particle size distribution of NiPd-30, with a particle size ranging from 1.47 µm to 16.66 µm and an average particle size of 6.32 µm. Compared to the sample after 10 h of ball milling, the average particle size of the sample in this stage decreases by approximately 64%. The difference between the maximum and minimum particle sizes narrows further. For NiPd-20, the particle size difference decreases from 69.09 µm to 35.98 µm. In the NiPd-30 sample, the proportion of particles smaller than 10 µm is approximately 91%, with a particle size difference of 15.19 µm. Hence, the particle size distribution becomes more uniform as ball milling time increases.

At this stage, the samples are primarily subjected to compressive and frictional forces, as illustrated in Figure 4a. This stage can be distinguished from the previous one and classified as the squeezing stage. Under compressive force, the relatively thick particles formed in the earlier stages (indicated by the blue box in Figure 4a) are continuously compressed axially and stretched radially, resulting in an overall increase in particle size and a decrease in thickness. Meanwhile, under the action of frictional force, small particles continuously flake off from the edges of the thin sheets, leading to surface roughening. As energy is continuously brought in, atomic migration becomes more pronounced, and the welding neck almost disappears. For metals with high hardness and brittleness, such as tungsten and its compound tungsten carbide, rapid fragmentation into smaller particles occurs. In contrast, more deformation takes place under stress for ductile metals such as nickel [30,31]. Since both Pd and Ni exhibit strong plastic deformation ability, dislocation lines form within the crystal lattice under external forces, resulting in slip bands. At the macroscopic particle level, this manifests as tensile deformation in a specific direction, extending up to tens of times without breaking. Therefore, many flaky particles are retained in this stage, leading to a significant reduction in the particle size distribution range compared to that in the previous stage, although it remains highly uneven. Further energy input is required to enhance the effects of squeezing and friction, so as to form smaller particles.

**Figure 7:**
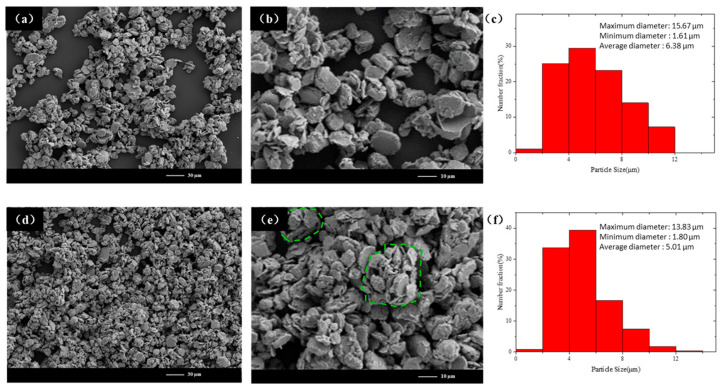
(**a**) Low-magnification SEM image and (**b**) high-magnification SEM image of NiPd-40. (**c**) Statistical graph of NiPd-40 particle size. (**d**) Low-magnification SEM image and (**e**) high-magnification SEM image of NiPd-50. (**f**) Statistical graph of NiPd-50 particle size. The spherical clusters are labelled within the green dashed lines.

As the ball milling process continues, the particle refinement process enters a new stage. The particle size of the alloy particles continues to decrease. Figure 7 shows the SEM images of the samples at this stage (NiPd-40 and NiPd-50). Figure 7a,b are low-magnification and high-magnification SEM images of NiPd-40, respectively. The particle distribution of the sample at this time is relatively uniform, and the particles that were axially superimposed in the previous stage have become smooth and compact. Figure 7c presents the particle size distribution of NiPd-40, with a particle size range from 1.61 μm to 15.67 μm and an average particle size of 6.38 μm. Figure 7d,e are low-magnification and high-magnification SEM images of NiPd-50, respectively. While the particle size is decreasing, the phenomenon of agglomeration is quite significant, as indicated by the green dashed box in the figure. The clusters are generally spherical, with disordered and randomly oriented particle stacking. Figure 7f shows the particle size distribution of NiPd-50, with a particle size ranging from 1.80 μm to 13.83 μm and an average size of 5.01 μm. Compared to the squeezing stage, the reduction in particle size in this stage is limited (about 21%). The particle size distribution at this stage is more uniform and concentrated in a certain range, with the particle size difference reduced from 15.19 μm to 12.03 μm.

The ball milling stage from 40 h to 50 h is characterized as the powder fragmentation stage. During this period, the effect of shear friction force on the sample particles becomes increasingly significant. Under the action of these forces, the corners of the flake-like particles in the radial direction continue to flake off, while transforming from irregular polygons to nearly circular. This transformation occurs because stress concentration at the vertices of the polygons leads to rapid fragmentation and the increasing of number of sides [32]. Studies have shown that the main factors affecting particle size during high-energy ball milling include the density and size of the grinding balls, the ball-to-powder ratio, and the speed of the ball milling. Additionally, time and the plastic deformation ability of the material influence the final particle shape [31]. Based on the input energy of system and the material intrinsic properties, it becomes challenging for the sample particles to further fragment into smaller sizes after this stage.

**Figure 8:**
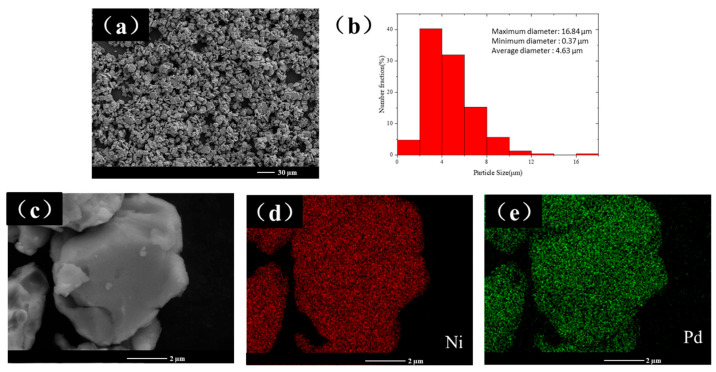
(**a**) Low-magnification SEM image of the NiPd-60 sample. (**b**) Particle size distribution histogram. (**c**) High-magnification SEM image. EDS mapping images of (**d**) Ni element and (**e**) Pd element.

To investigate the alloying process of Ni and Pd during ball milling, we characterized the elemental distribution of the milled samples. Figure 8 shows the experiment results of the NiPd-60 sample. As shown in Figure 8a, both the size distribution and geometric outlook of the sample are relatively uniform, and the particles show a near round morphology. The particle size distribution histogram in Figure 8b indicates that the sample consists of flake-like particles with a radial size of approximately 4.63 μm and an axial size of approximately 1 μm. Particles with a size smaller than 6 μm account for about 76.8% of the total, while those smaller than 4 μm account for 45%. After 60 h of ball milling, the average particle diameter of the NiPd alloy is reduced by approximately 10 times, with an average value of 4.63 μm. Some small particles with a minimum size of 170 nm denoted by the yellow arrow are attached to the larger alloy particles, constituting a sample with a mixture of micro- and nano-sized particles at dual scales. The magnified SEM image (Figure 8c) shows that the particle surface is relatively smooth, with smaller debris adhering to it. Elemental analysis (Figure 8d,e) reveals that the two elements are distributed very uniformly, indicating complete alloying of the sample.

After the fragmentation stage, the energy provided by the impact and shear friction force within the system under this ball milling parameter has reached its maximum. The alloy particles are simultaneously forming new particles through cold welding and breaking into smaller particles under the action of various forces, achieving a dynamic balance. The particle size remains relatively stable within a certain range. Due to the strong plastic deformation ability of metals Pd and Ni, it is difficult to break into smaller particles. At a rotational speed of 500 rpm, the size of most of the particles cannot be reduced to the nanoscale with further increasing the ball milling time. Instead, only some debris that has been impacted or rubbed off crush into the nano-sized particles. Increasing of the ball milling time leads to particle aggregation and an increase in particle size. To reduce the particle diameter, measures such as adjusting the ball-to-powder ratio, increasing the rotational speed, and changing the size of grinding balls can be taken.

**Figure 9:**
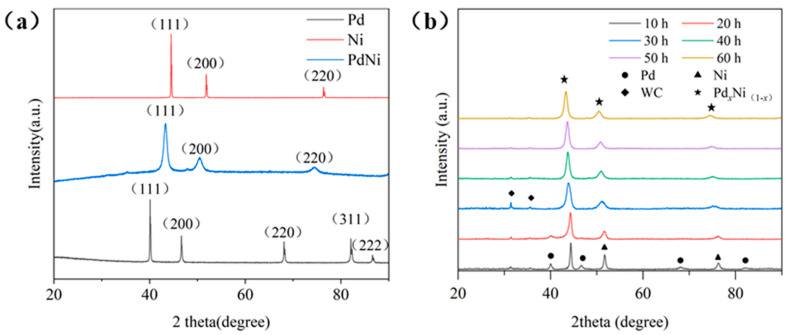
(**a**) XRD patterns of the initial and ball-milled alloy samples. (**b**) XRD patterns of samples with different ball milling times.

In addition, XRD patterns were conducted on the initial samples and samples at different ball milling process. Figure 9 show the results of XRD patterns. Figure 9a presents the XRD patterns of the initial elemental powders and the alloy sample after 60 h of ball milling. In comparison, it is evident that the peaks of elemental Ni and Pd have almost disappeared after 60 h of ball milling. The three peaks in the alloy XRD pattern correspond to different NiPd alloy phases with the main crystal plane being the (111) plane. The peak height of the alloy phase is relatively low, and the full width at half maxima (FWHM) is relatively wide, showing appreciable changes compared to the sharp and high peaks in the elemental patterns. This indicates that the alloying of the sample has been completed using the HEBM and that the particle size has decreased significantly. Figure 9b shows the XRD patterns of samples at different ball milling times. At 10 h of ball milling, the peaks of elemental Pd and Ni still exist. However, their intensities have been reduced, and the FWHM is broadened compared to the initial state. Simultaneously, new phases have formed. These results indicate that at this stage, the particles for the elementary substances become smaller under the action of impact and friction forces, resulting in a broadening of the FWHM of the elemental peaks. Additionally, new peaks appear with a narrower FWHM, corresponding to particles that have begun to weld and pre-alloy. These changes are consistent with the SEM image analysis presented earlier. As ball milling continues and noted in the pattern at 30 h, all elemental phases have disappeared, indicating that all elementary substances have entered alloying after the squeezing stage. However, the peak positions shift slightly compared to the peaks in final alloy phase, indicating incomplete alloying at this stage. After this stage, as ball milling continues, the changes in the sample patterns are not obvious. On the one hand, the position of the alloy phase continuously shifts to the left, indicating more complete alloying of the sample. On the other hand, there is a slight broadening in the FWHM, corresponding to the particle size changes shown in Figure 3. Additionally, a small amount of WC phase components were detected in the XRD patterns. This is because the vials and grinding balls used in the ball milling process are made of WC, and a small amount of WC had mixed into the sample during the ball milling process.

### 3.3. The Influence of the Ball-to-Powder Ratio on the Ball Milling Process of NiPd Alloy

**Figure 10:**
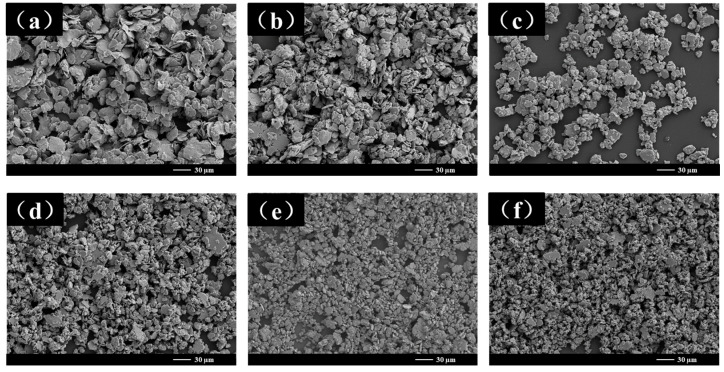
SEM images of NiPd samples of BPR20 with different ball milling times: (**a**) 10 h; (**b**) 20 h; (**c**) 30 h; (**d**) 40 h; (**e**) 50 h; (**f**) 60 h.

In the previous section, the changes in the grain size of NiPd samples during ball milling were discussed, and a model for grain refinement was proposed. During the ball milling process, the samples primarily undergo four stages: welding and deformation, squeezing, fragmentation, and dynamic balance. As mentioned earlier, under the determined metallic properties and ball milling parameters, the outcome of ball milling is predictable. The ball-to-powder ratio is one of the most critical parameters for controlling the structure and morphology of the samples. Studies have shown that the BPR significantly impacts the efficiency of ball milling [33]. To further investigate the influence of different BPRs on the ball milling process and the structural and morphological features of NiPd alloy samples, experiments with different BPRs were conducted. Specifically, experiments were carried out with BPRs of 10:1 (labeled as sample BPR10) and 20:1 (labeled as sample BPR20), and the SEM images of the samples at different times are shown in Figure 2 and Figure 10, respectively. Compared with the BPR10 samples analyzed in detail in the previous text, the ball milling stages experienced by the BPR20 samples are similar to those of the BPR10 samples. Both samples in these two regimes start from an initial two-dimensional flake-like structure and gradually transform into smaller radial dimensions, smaller-sized particle structures, and dual-scale polycrystalline structures. The differences between the BPR10 and BPR20 need to be analyzed through particle size statistics. The particle size of the sample at each stage in various BPRs was statistically analyzed three times, and the average value was calculated as the final result.

**Figure 11:**
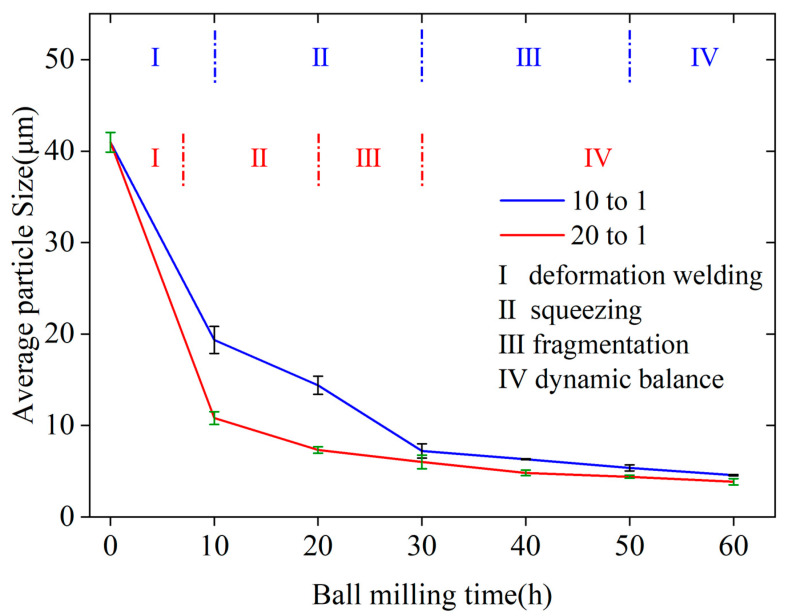
Variation in the average particle size of the sample over time with different BPRs.

Figure 11 shows the trend in particle size variation over time for two BPRs. Similar to BPR10, the samples of BPR20 also undergo the same changes during the ball milling process: thinning and expansion, axial overlap and thickening, surface roughening followed by smoothing, and eventual rounding. Although the overall ball milling process is similar, there are noticeable differences in the samples at different times and BPRs. By comparing the results of BPR10 and BPR20, it can be seen that they exhibit distinct characteristics. After 10 h of ball milling, the particle size distribution of the BPR10 sample is uneven, with an average particle size of 17.72 µm, and mostly large and thin plate-like structures with relatively smooth surfaces are observed. In contrast, the particle size distribution of the BPR20 is more uniform, with an average particle size of 10.30 µm, approximately 58% of that of BPR10. The surface of the BPR20 particles is relatively rough and has a certain thickness in the radial direction, resembling the particle morphology in the squeezing stage of BPR10. The particles have been transformed into smaller ones under the action of friction and impact, reaching about 168% of the size at the end of the squeezing stage of BPR10, indicating that they have entered the intermediate stage of the squeezing stage. After 20 h of ball milling, the particle size of the BPR20 sample is comparable to that of the BPR10 sample after 30 h of ball milling, and the subsequent reduction in particle size slows down. This indicates that the squeezing stage is completed 10 h earlier when the ball-to-powder ratio is doubled. When the ball milling time is 30 h, the particle size of the BPR20 sample is 5.52 µm. This is comparable to that of the BPR10 sample at a ball milling time of 50 h, which suggests that it has completed the fragmentation stage and entered the dynamic balance stage. The results indicate that BPR20 completes the first three stages of the ball milling process earlier compared with BPR10, significantly improving ball milling efficiency by saving 20 h. After 60 h of ball milling, the average particle size of BPR20 is 3.62 µm, which is 22% lower than that of the BPR10 sample, showing an insignificant reduction. On the other hand, from Figure 10f, it can be seen that compared with the BPR10 samples at the same time, there are individual large particles due to particle aggregation caused by an excessive ball milling time, which should be avoided as much as possible. Finally, when the loading amount is constant during the ball milling process, increasing the ball-to-powder ratio improves efficiency but reduces output production. Factors such as preparation quantity and the aforementioned ball milling parameters should be selected comprehensively in the actual preparation process.

Research demonstrates that increasing the BPR not only significantly accelerates the progress of the ball milling process but also reduces the final particle size of the sample. The increase in the BPR can be achieved by increasing their numbers while keeping the ball size constant. An increase in BPR leads to an increased contact area between the grinding balls and the sample, resulting in a sharp increase in the number of impacts between the grinding balls and powder particles, as well as a more thorough application of impact force and shear friction. Some researchers revised the model proposed by A.S. Kurlov and A.I. Gusev for planetary ball milling [34,35], incorporating the number of balls *N_b_* and their mass *m* into the BPR. Thus, Equation (1) can be used to describe the influence of BPR on the particle size of NiPd powder at different time durations [36]:

(1) $$D\left(t,M,BPR\right)=\frac{\left\{A+\;B\left[\ln\left(D_{in}/2b\right)\right]\varepsilon_{max}\left[t/\left(t+\tau \right)\right]\left[1/\left(1+p/M\right)\right]\right\}}{\kappa^{\prime }\omega^{3}t\cdot BPR+\left\{A+B\left[\ln\left(D_{in}/2b\right)\right]\varepsilon_{max}\left[t/\left(t+\tau \right)\right]\left[1/\left(1+p/M\right)\right]\right\}/D_{in}},$$

(2) κ′ = 8π3akRC2+r212RC64−3r/RC464−16r/RC2,

(3) $$\varepsilon =\;\varepsilon_{max}\left[t/\left(t+\tau \right)\right]\left[M/\left(M+p\right)\right],$$

where A and B are constants that describe the characteristics of the powder, ***b*** is the Burgers vector, *κ* is a constant related to the ball mill, *r* is the radius of the vial, *R*_c_ is the radius of the circle along which the vial axis moves, *a*_k_ represents the energy coefficient consumed by the powder grinding, *D*_in_ is the average initial particle size of the powder, *M* is the initial mass of the powder, *D*(*t*,*M*,*BPR*) is the final particle size, *τ* and *p* are normalized parameters, *ω* is the rotational angular velocity of the ball mill, *t* is the ball milling time, and *ε* is the micro-strain dependent on time and initial mass. Given that the material is fixed and the ball mill parameters remain unchanged in this study, Equation (1) can be simplified as follows:

(4) $$D\left(t,M,BPR\right)=\frac{\left[C_{1}+\;C_{2}\cdot \varepsilon \left(t,M\right)\right]}{C_{3}\cdot t\cdot BPR+\left[C_{1}+C_{2}\cdot \varepsilon \left(t,M\right)\right]/D_{in}},$$

where *C*_1_, *C*_2_, and *C*_3_ are constants. Furthermore, since *p* is very small, the influence of the initial powder mass on the micro-strain can be neglected. Therefore, Equation (4) can be simplified as follows:

(5) $$D\left(t,BPR\right)=\frac{\left[C_{1}+C_{2}\cdot \varepsilon_{max}\left[t/\left(t+\tau \right)\right]\right]}{C_{3}\cdot t\cdot BPR+\left[C_{1}+C_{2}\cdot \varepsilon_{max}\left[t/\left(t+\tau \right)\right]\right]/D_{in}},$$

It can be seen from Equation (5) that when the ball milling time is constant, the particle size decreases with an increase in BPR. This observation aligns with the differences in particle size at the same milling time but different BPRs shown in Figure 11. When the initial sample and ball milling conditions are identical, a larger BPR results in a smaller particle size. On the other hand, when BPR is fixed, the micro-strain exhibits a trend of a rapid increase followed by stabilization as ball milling time increases. Similarly, the particle size rapidly decreases and then tends to reach a constant value with increasing milling time, which is consistent with the experimental results.

## 4. Conclusions

NiPd alloy powder with a two-dimensional flake-like structure and micro-nano dual sizes was successfully synthesized using HEBM. To the best of our knowledge, this is the first time such a structure has been achieved using HEBM, a simple yet highly effective method. The dual sizes of NiPd alloy powder under the condition of high BPR reach 3.6 μm and 158 nm. Additionally, the mechanism of the particle refinement process and the influence of the BPR were elucidated. The ball milling process of NiPd alloy materials generally includes the following stages. Single-element particles become fragmented and thinner under impact forces in the deformation and welding stage, and some particles are welded in both radial and axial directions. The radial diameter increases beyond the original size, and the NiPd particles gradually form a flake-like microstructure and undergo thickening after thinning. Radially superimposed particles in the radial direction become increasingly compact in the squeezing stage, leading to continuous alloying and intensified atomic movement. Flake-like particles are gradually crushed into smaller particles in the fragmentation stage. Due to the limits of input energy from ball milling, the particle size fluctuates within a certain range, reaching a dynamic balance between fragmentation and recombination in the dynamic balance stage. The study on the BPR indicates that a higher BPR can significantly accelerate the ball milling process to reach an efficient crushing. This study elucidates the influence of ball milling time and BPR on the microstructure of NiPd alloy, thereby addressing a critical gap in the preparation of NiPd alloy using HEBM. Furthermore, the technique and physical mechanisms explored in this study exhibit the capacity for transplantation, providing valuable guidance and strategies for the practical application in the fabrication of NiPd alloy and other types of metal alloys in the future.

## Data Availability

The original contributions presented in this study are included in the article. Further inquiries can be directed to the corresponding author.
